# Factors associated with health facility childbirth in districts of Kenya, Tanzania and Zambia: a population based survey

**DOI:** 10.1186/1471-2393-14-219

**Published:** 2014-07-04

**Authors:** Selia Ng’anjo Phiri, Torvid Kiserud, Gunnar Kvåle, Jens Byskov, Bjørg Evjen-Olsen, Charles Michelo, Elizabeth Echoka, Knut Fylkesnes

**Affiliations:** 1 Centre for International Health, Department of Global Health and Primary Care, University of Bergen, Bergen, Norway; 2 Department of Obstetrics and Gynaecology, Haukeland University Hospital, Haukeland, Norway; 3 Department of Clinical Science, University of Bergen, Bergen, Norway; 4 Centre for Health Research and Development, Faculty of Health and Medical Sciences, University of Copenhagen, Copenhagen, Denmark; 5 Department of Obstetrics and Gynaecology, Sørlandet Hospital, Flekkefjord, Norway; 6 Department of Public Health, School of Medicine, University of Zambia, Lusaka, Zambia; 7 Centre for Public Health Research, Kenya Medical Research Institute, Nairobi, Kenya

**Keywords:** Health facility childbirth, Home deliveries, Socioeconomic position, Inequity, Africa, Kenya, Tanzania, Zambia

## Abstract

**Background:**

Maternal mortality continues to be a heavy burden in low and middle income countries where half of all deliveries take place in homes without skilled attendance. The study aimed to investigate the underlying and proximate determinants of health facility childbirth in rural and urban areas of three districts in Kenya, Tanzania and Zambia.

**Methods:**

A population-based survey was conducted in 2007 as part of the ‘REsponse to ACcountable priority setting for Trust in health systems’ (REACT) project. Stratified random cluster sampling was used and the data included information on place of delivery and factors that might influence health care seeking behaviour. A total of 1800 women who had childbirth in the previous five years were analysed. The distal and proximate conceptual framework for analysing determinants of maternal mortality was modified for studying factors associated with place of delivery. Socioeconomic position was measured by employing a construct of educational attainment and wealth index. All analyses were stratified by district and urban–rural residence.

**Results:**

There were substantial inter-district differences in proportion of health facility childbirth. Facility childbirth was 15, 70 and 37% in the rural areas of Malindi, Mbarali and Kapiri Mposhi respectively, and 57, 75 and 77% in the urban areas of the districts respectively. However, striking socio-economic inequities were revealed regardless of district. Furthermore, there were indications that repeated exposure to ANC services and HIV related counselling and testing were positively associated with health facility deliveries. Perceived distance was negatively associated with facility childbirth in rural areas of Malindi and urban areas of Kapiri Mposhi.

**Conclusion:**

Strong socio-economic inequities in the likelihood of facility childbirths were revealed in all the districts added to geographic inequities in two of the three districts. This strongly suggests an urgent need to strengthen services targeting disadvantaged and remote populations. The finding of a positive association between HIV counselling/testing and odds in favor of giving birth at a health facility suggests potential positive effects can be achieved by strengthening integrated approaches in maternal health service delivery.

## Background

Maternal mortality continues to be a burden in low and middle income countries where 60% of deliveries take place outside health facilities, and skilled personnel assist only half of all deliveries [1,2]. Although a 47% decline from 1990 was reported, 287,000 women still died from pregnancy related complications in 2010 [3]. Low and middle income countries accounted for 99% of these maternal deaths, and sub-Saharan Africa alone for 56% [3]. The life-time risk of maternal death is 1/39 in sub-Saharan Africa, compared with 1/3800 in high income countries [3].

The majority of maternal deaths arise from complications that are not predicted during the antenatal period, but occur just before, during or after birth [4]. Therefore, skilled birth attendance (SBA) becomes crucial for effective intervention to save both women and neonates during childbirth. It is estimated that SBA could reduce by 16-33% maternal deaths due to haemorrhage, obstructed labour, puerperal sepsis and preeclampsia [5] and 39-71% deaths of neonates related to birth asphyxia during intra-partum period [6], presuming that facilities meet standards of quality care. Skilled attendance comprises skilled personnel, an organised enabling delivery environment, adequate supplies and equipment, and efficient communication and transport for referrals [5]. A skilled birth attendant is someone ‘trained to proficiency in the skills needed to manage normal pregnancies, childbirth and immediate postpartum period, and in the identification, management and referral of complications in women and newborns’ [7]. An enabling environment is one that provides Emergency Obstetric and Newborn Care (EmONC) services. Basic EmONC services include administering parenteral antibiotics, uterotonic drugs, and anticonvulsants, manual removal of retained placenta and removal of retained products of conception, perform assisted vaginal delivery using vacuum extraction, forceps delivery, and perform basic neonatal resuscitation. Comprehensive EmONC services include basic EmONC as well as surgery and provide blood transfusion [8]. Inadequacies in competence of health personnel and provision of EmONC services have been observed in several countries with high maternal mortality rates [9], which could retard the intended benefits of SBA.

In sub-Saharan Africa, delivery care seems not to have improved significantly the past decades [3,10]. Although benefits of SBA are well known and the Safe Motherhood Initiative had already started in 1987, the Millennium Development Goal (MDG) five of reducing maternal mortality by three quarters by 2015 seems remote [11]. Several factors contribute to lack of progress in sub-Saharan Africa. Women’s education, household wealth [12-14] and health status of the women, especially with the serious HIV epidemic, are examples of such factors [3,15]. Inadequate availability and distribution of skilled providers and emergency obstetric care, suboptimal standards, cost of services, transportation and road infrastructure are further barriers [12,16-18]. The greatest inequity in healthcare seems to occur in maternal and newborn health [19], especially with skilled attendance at birth [20]. Equity in health, which is an ethical concept, is defined as ‘the absence of systematic disparities in health between social groups who have different levels of underlying social advantage/ disadvantage’ [21]. Health inequity is ‘those inequalities that are not only unnecessary and avoidable but, in addition, are also unfair and unjust’ and indicates that what the socially advantaged groups have achieved is also attainable for others [22].

Kenya, Tanzania and Zambia, like the rest of sub-Saharan Africa, have shown little improvement in maternal mortality and skilled attendance at birth to date [23,24]. In Zambia, despite a reduction of direct obstetric causes of maternal mortality observed in a tertiary referral hospital, the overall mortality did not improve due to increasing non-obstetric causes, mainly malaria and HIV/AIDS [25]. Estimated proportions of skilled attendance at delivery have remained low, with 44, 51 and 47% in Kenya, Tanzania and Zambia, respectively [26-28]. Rural and urban differences exist, with Kenya reporting 75% women in urban and 37% in rural receiving SBA; Tanzania 83% and 42% in urban and rural areas respectively; Zambia 83% in the urban and 31% in rural areas [26-28]. Although much is known about contributing factors, many gaps remain in our understanding of how to deliver interventions that would bring about improved provision and utilization of skilled attendance in resource limited settings with weak health systems.

Systematic priorities need to guide decisions on the type of interventions in limited healthcare resource settings. Accountability for Reasonableness (AFR) is an ethical framework, which has been proposed to guide achievement of a fair and legitimate priority setting process [29]. Fair and legitimate priority setting in health systems could guide stakeholders to establish priorities that enhance trust, quality and equity. This study used baseline data from the Response to Accountable priority setting for Trust in health systems (REACT) research project, a multicentre study entitled “*Strengthening fairness and accountability in health systems priority setting for improving equity and access to quality healthcare at district level in Tanzania, Kenya and Zambia”*[29]. The REACT research project, which uses an AFR framework, focuses on introducing and evaluating the outcomes of an approach to fairness in priority setting within health systems management, human resources, HIV/AIDS control, emergency obstetric care, malaria control, and generalised care. A comparison across countries was undertaken as a basis for designing setting specific and context driven interventions. This study investigated factors associated with health facility childbirth in rural and urban areas in the three districts.

## Methods

### Study site

The survey was conducted in 2007. Selected sites were Malindi in Kenya, Mbarali in Tanzania, and Kapiri Mposhi in Zambia. The sites were selected based on the assumption of their similarities in disease burden and health systems [29], although, the organization of the healthcare system in the three districts appears to differ substantially [30]. The estimated population in the study areas was 350,000 in Malindi, 235,000 in Mbarali and 200,000 in Kapiri Mposhi [31-33].

Malindi is located in the Coast Province along the Kenyan border with the Indian Ocean. It is a major tourist destination, and its main economic activities include fishing, forestry, tourism, and agriculture. Malindi has a crude birth rate of 48 per 1,000, growth rate of 3.9%, and total fertility rate (TFR) of 6.1, with contraceptive acceptance rate of 29% [31]. It has three hospitals (one government and two private) and 24 dispensaries (17 government and seven non-governmental organizations) [31]. Mbarali is situated in the Mbeya region of Tanzania. It is along the major road connecting Mbeya city with Dar es Salaam, and a railway line that joins Kapiri Mposhi in Zambia to Dar es Salaam. The main economic activity is agriculture. The district’s growth rate is 2.8%, and the TFR for the southern region is 4.4 [27]. Mbarali has two hospitals in Chimala and Rujewa, two health centres and 39 dispensaries. Most of the health facilities are supported by health insurance services from government, National Health Insurance Fund [34]. In contrast and similarities, Kapiri Mposhi is in the Central Province and a gateway to the north of the country via road and railway connections. The main economic activity is agriculture. The TFR for Central Province is 6.4 [28]. The crude birth rate is 43 per 1,000 and a growth rate of 2.7% [33]. Kapiri Mposhi has 25 public health centres, two health posts, one private and two mission health centres [35]. A second level referral hospital located about 50 kilometres south of the district in Kabwe, served referrals from Kapiri Mposhi until 2011 when a new hospital was opened within the district.

### Study design

The data stem from a population-based survey employing multi-stage stratified random cluster design. Stratification was by district and within district by rural–urban residence. Rural and urban were defined according to population censuses of 1999, 2002 and 2000 in Kenya, Tanzania and Zambia, respectively [36]. A combination of politico-administrative perspective and human settlements perspective were used with Kenya defining urban as municipalities with 2,000 inhabitants or more; whereas Tanzania defined this by size and density with majority of their inhabitants in non-agricultural occupations; and Zambia defining urban as localities of 5,000 inhabitants or more and a majority of the labour force in non-agricultural activities [36]. Proportions of urban population were 21, 23 and 35% for Kenya, Tanzania and Zambia respectively [26-28]. Standard enumeration areas (SEAs) were used as basic sampling units. First, clusters that corresponded with the SEAs at the district were selected using probability proportional to size. The listing of SEAs provided information on households based on the population census. A total of 49 clusters (Malindi 10, Mbarali 19, Kapiri Mposhi 20) were selected from the urban stratum, and 70 clusters (Malindi 19, Mbarali 26, Kapiri Mposhi 25) from the rural stratum. The second stage involved randomly selecting a fixed number of households from the list that was compiled consisting of all households in the selected SEAs in each district. The aim was to select 2,000 individuals in each district. One male and one female aged between 15 and 49 years of age were randomly selected as participants in each household. This study analysed women respondents who had delivered within five years prior to the study and information obtained about the most recent childbirth.

### The conceptual framework

A conceptual framework by McCarthy and Maine [37] was employed to guide analysis. The framework guides analysis of maternal mortality determinants and could be applied to research and programmes [37]. The concept grouped determinants as distant factors that are underlying socio-economic and cultural factors; intermediate or proximate factors, such as healthcare seeking behaviour and use of health services, health status, and access to services, which directly influence pregnancy outcomes of morbidity and mortality. The distal socio-economic and cultural factors are mediated through the health seeking behaviour and access to health care service to result in pregnancy outcome [37]. Considering evidence from studies that relate EmONC services to reduced maternal mortality [38], we found this framework applicable to our study on use of health facilities for childbirth, with the presumption that facilities provide skilled birth attendance and EmONC services.

Selection of variables to include in our model was guided by previous studies and the above framework. It was hypothesized that underlying socioeconomic position, age and marital status were associated with health facility childbirth. Women’s educational attainment and wealth status have been associated with health facility childbirth in sub-Saharan Africa and other regions [12-14,39,40]. Single women seem to have more autonomy than married women, and conversely young women may not have the financial capability to access health services [41]. In sub-Saharan Africa, older age has been associated with reduced health facility childbirth [16,42]. It was hypothesized that proximate factors of access to health services measured by perceived distance and perceived cost were associated with reduced health facility childbirth, as observed in previous studies [16-18,43]. We also hypothesized that trust and perceived quality of care, use of health care services during ANC visits and exposure to HIV counselling and testing were associated with increased health facility childbirth. Perceived quality of care has been associated with by-pass of the nearest health facility, and ANC visits with increased use of health facility at delivery [16,18,44-46].

### Data collection and variables

Data was collected in 2007 by trained enumerators using a structured questionnaire. The data collection tools were developed and standardised for application in the three countries within a standard operating procedure for training of staff, and pilot testing of the tools. EpiData version 3.1 was used for data entry.

The dependent variable was place of childbirth dichotomised as: home delivery = 0, all health facility deliveries = 1. Only women having given birth the previous five years or less were included in the analysis. Independent underlying variables included socioeconomic position (SEP), age and marital status categorised as single (never married, widowed, separated and divorced), and married (married, cohabitating). SEP was created by summation of wealth index and the woman’s educational attainment in school years. Wealth index was a summation of information on electricity; asset ownership of radio, television, refrigerator, bicycle, plough, donkey, cattle; and type of housing construction material. SEP is seen as a multidimensional and multilevel construct which is partly determined by structural relations. We used educational attainment and wealth index as indicators of SEP with the intention to capture the association between facility childbirth and SEP as part of the assessment for equity. SEP was categorised as low, middle and high to indicate proportions of facility childbirths but retained as a continuous variable in the model. Educational attainment and wealth index were also analysed as separate variables in the model to compare with the model using SEP variable. However, only educational attainment remained as a significant factor. Since the two indicators were highly correlated (r = 0.2), we used the composite variable in analysis. Proximate variables included a composite “trust-quality”, which was created using self-rating of local health services, perceived drug availability and perceived attitudes of the health care staff at the nearest clinic. These three variables were correlated and principal component analysis was used for data reduction. The trust-quality variable was categorized into four groups ranging from ‘very bad’ to ‘very good’ and later condensed the adjacent groups to create two categories, ‘bad’ and ‘good’. The non-categorized variable was retained and used as a continuous variable in the model. Other proximate variables were perceived cost and perceived distance which were categorized using a Likert scale ranging from “not at all” to “very much” and later condensed as “not at all/little”, “fairly” and “much/very much” while maintaining the uncondensed variable as a continuous quantity in the model. The number of ANC attendance and ever tested for HIV (yes, no) were used as proxies to use of health services during pregnancy. ANC attendance was grouped as 0–3 visits, 4 or more visits, and was used as continuous in the model.

### Data analysis

Data analysis was done using SPSS for Windows Version 19, SPSS Inc. Chicago, Illinois. Descriptive statistics and multivariate analysis were done and complex sample design used to take into consideration the design effect. Stepwise multivariate logistic regression was used to estimate adjusted associations. In step1 only the underlying (or distal) factors were included in the model, whereas in step 2 the proximate factors were also included. The variables selected in the model were those that were found significant in bivariate analysis in any of the three districts. Analyses were stratified by district and by rural–urban residence.

### Ethical clearance

Ethical clearance was obtained in Kenya from Kenya Medical Research Institute (KEMRI) and from the National Ethical Review Committee (NERC); in Tanzania from the Medical Research Coordinating Committee (MRCC) of the National Institute of Medical Research (NIMR); and in Zambia the University of Zambia Research Ethics Committee. Written informed consent was obtained from all participants of the population based surveys prior to being interviewed. Confidentiality and anonymity of the study participants was maintained. This study was specifically approved by the Steering Committee of REACT, which is also the project review board including representatives from all three study countries.

## Results

### Population characteristics

The number of women analysed was 1800, with 583 from Malindi, 687 from Mbarali and 530 from Kapiri Mposhi. The overall response was 93.4%. Their age ranged from 15 to 49 years with mean of 28.5 ± 6.8 SD in Malindi, 28.2 ± 6.3 SD in Mbarali, and 28.9 ± 7.5 SD in Kapiri Mposhi. Median age was 28.0 for all the districts. Non-response did not differ by district or socio-demographic and socio-economic characteristics.

### Place for childbirth

Significant differences in health facility childbirth between rural and urban areas of Malindi and Kapiri Mposhi, p < 0.001, and in place for childbirth in all the three districts, p < 0.001, were observed. No significant rural urban difference was observed in Mbarali, p = 0.156. In Malindi, 85% and 43% delivered at home in rural and urban areas respectively, with 40% in a public hospital in urban areas. In Mbarali, 30% and 25% had childbirth at home in rural and urban areas respectively, with 37% in public health centres in rural areas, and 29% in mission health facilities in urban areas. In Kapiri Mposhi, 63% and 23% delivered at home in rural and urban areas respectively. Use of a public hospital was 18% and 67% in rural and urban areas respectively (Table 1).

**Table 1:** **Distribution by place (rural versus urban) and type of facility for childbirth in women aged 15–49 years in districts of Malindi, Mbarali and Kapiri Mposhi (N = 1800) **^**§**^

**Place of delivery**	**Malindi**			**Mbarali**			**Kapiri Mposhi**		
	**Rural (%)**	**Urban (%)**	*p-value	**Rural (%)**	**Urban (%)**	*p-value	**Rural (%)**	**Urban (%)**	*p-value
Home	84.7	43.0	<0.001	30.0	25.2	0.156	63.2	22.9	<0.001
Public health centre/dispensary	6.7	5.5		37.0	20.7		14.0	10.0	
Public hospital	7.2	40.0		13.1	17.5		17.8	67.1	
Private health facility	1.2	9.7		2.1	6.7		-	-	
Mission/NGO health facility	-	1.8		14.2	29.0		5.0		
Other	0.2	-		3.5	1.0		-	-	
Total (n)	418	165		373	314		299	231	

*Pearson’s chi square test for independence between rural and urban areas χ^2^ = 103.55, df = 1, p <0.001 for Malindi, χ^2^ = 84.55, df = 1, p < 0.001 for Kapiri Mposhi, and χ^2^ = 2.01, df = 1, p =0.156 for Mbarali.

^§^Women with childbirth in the previous five years before and until 2007.

Significant differences in health facility childbirth were also observed across districts, p < 0.001. The prevalence ratio of facility childbirth in Mbarali compared to Malindi was 4.6 in rural areas and 1.3 in urban areas. Comparing Kapiri Mposhi to Malindi the prevalence ratio of facility childbirth was 2.4 in rural areas and 1.4 in urban areas (Table 1).

### Facility childbirths with underlying factors

#### Socio-demographic factors

Married women in urban areas of Kapiri Mposhi tended to be more likely to have facility childbirth compared to single women, and this association remained significant after adjusting for SEP and proximate factors (Tables 2 and 3). In Mbarali the number of single women was very small. Place of delivery did not differ by age. There was no significant difference with age observed in the final model in all three districts (Tables 3, 4 and 5).

**Table 2:** **Frequencies and proportions of health facility childbirths by socio-economic, socio-demographic and proximate factors in rural and urban areas of Malindi, Mbarali and Kapiri Mposhi in women aged 15–49 years (N = 1800)**^**§**^

	***MALINDI***				***MBARALI***				***KAPIRI MPOSHI***			
	**Frequency n**		**Proportion of facility childbirths (%)**		**Frequency n**		**Proportion of facility childbirths (%)**		**Frequency n**		**Proportion of facility childbirths (%)**	
	**R†**	**U‡**	**R**	**U**	**R**	**U**	**R**	**U**	**R**	**U**	**R**	**U**
*Underlying factors#*	n = 418	n = 165			n = 373	n = 314			n = 299	n = 231		
**SEP**												
Low	239	29	11.3	37.9	114	81	64.0	70.4	141	59	26.4	72.9
Middle	106	43	19.8	41.9	124	112	65.3	67.0	74	68	45.9	76.5
High	73	93	21.9	69.9	135	121	79.3	85.1	84	104	46.4	79.8
**Age**												
15 - 19 years	29	18	24.1	55.6	18	22	55.6	77.3	27	19	29.6	78.9
20-29 years	204	89	17.7	53.9	206	167	70.4	77.8	140	122	39.3	75.4
30+ years	185	58	11.4	62.1	149	125	71.1	70.4	132	89	35.9	78.7
**Marital status**												
Married	378	128	15.6	55.5	366	302	69.7	74.8	250	185	37.8	80.0
Single/Divorced	40	37	12.5	62.2	7	12	85.7	75.0	49	46	32.7	65.2
***Proximate factors##***												
**Trust-Quality**												
Bad	148	64	14.9	57.8	247	177	72.9	68.4	154	134	35.1	76.1
Good	258	97	16.0	54.6	107	114	67.3	87.7	137	83	40.4	79.5
**Perceived cost**												
Not at all/little	140	70	19.3	68.6	229	88	71.6	62.5	191	156	36.6	74.4
Fairly	161	47	15.0	48.9	43	52	72.1	73.1	38	35	47.4	82.9
Much/ very much	110	48	10.9	47.9	98	168	67.3	83.3	69	39	30.9	82.1
**Perceived distance**												
Not at all/little	131	124	26.0	57.3	225	130	73.8	73.1	135	160	43.0	76.9
Fairly	120	34	16.0	58.8	36	47	72.2	83.0	28	24	28.6	83.3
Much/ very much	161	7	6.2	42.9	110	131	61.8	75.6	135	46	32.1	73.9
**ANC visits**												
0-3 visits	179	61	9.6	47.5	140	110	64.3	72.7	102	70	29.7	68.6
4+ visits	239	104	19.7	62.5	233	204	73.4	76.0	197	161	40.6	80.7
**Ever HIV tested**												
Yes	258	105	16.3	61.9	120	94	75.8	87.2	112	127	44.6	81.1
No	158	60	14.0	48.3	253	218	67.2	69.3	185	101	32.6	73.3

^§^Women with childbirth in the previous five years before and until 2007 were included.

†R Indicates rural.

‡U Indicates urban.

^#^Total numbers in urban Kapiri Mposhi for age variable do not add up to 231 due to one missing value.

^##^Total numbers in the different categories of proximate factors do not add up to the sub-total ‘n’ due to missing values.

**Table 3:** **Bivariate and multivariate logistic regression of health facility childbirths adjusted by socio-economic, socio-demographic and proximate factors in Kapiri Mposhi district, Zambia in women aged 15–49 years (N = 1800)**^**§**^

	**Bivariates**				**Multivariate step 1**				**Multivariate step 2 (full model)**			
	**Rural**		**Urban**		**Rural**		**Urban**		**Rural**		**Urban**	
	**OR (95% CI)**	**p value**	**OR (95% CI)**	**p value**	**AOR (95% CI)**	**p value**	**AOR (95% CI)**	**p value**	**AOR (95% CI)**	**p value**	**AOR (95% CI)**	**p value**
**Underlying factors**												
**SEP (1–16)**†	**1.14 (1.07-1.22)**	**<0.001**	**1.10 (1.02-1.18)**	**0.009**	**1.14 (1.07-1.22)**	**<0.001**	**1.10 (1.02-1.18)**	**0.011**	**1.14 (1.07-1.21)**	**<0.001**	**1.10 (1.04-1.17)**	**0.003**
**Age (15–49)**	1.00 (0.98-1.03)	0.898	0.99 (0.94-1.04)	0.620	1.00 (0.97-1.03)	0.890	0.99 (0.94-1.04)	0.662	1.00 (0.97-1.04)	0.831	1.00 (0.94-1.07)	0.914
**Marital status**												
Married	1.00	0.448	1.00	**0.022**	1.00	0.308	1.00	**0.020**	1.00	0.452	1.00	**0.024**
Single/Divorced	0.80 (0.45-1.43)		**0.47 (0.25-0.89)**		0.75 (0.42-1.33)		**0.45 (0.24-0.86)**		0.80 (0.43-1.47)		**0.43 (0.21-0.88)**	
**Proximate factors**												
**Trust-Quality (1–4)**	1.12 (0.91-1.37)	0.293	1.12 (0.82-1.54)	0.465					1.15 (0.94-1.40)	0.157	1.11 (0.79-1.57)	0.553
**Perceived cost (1–5)**	0.96 (0.81-1.13)	0.549	1.19 (0.95-1.49)	0.130					1.01 (0.84-1.23)	0.916	**1.34 (1.02-1.77)**	**0.039**
**Perceived distance (1–5)**	0.89 (0.76-1.05)	0.135	0.97 (0.83-1.13)	0.658					0.85 (0.72-1.01)	0.053	**0.77 (0.66-0.90)**	**0.002**
**ANC visits (0–9)**	1.17 (0.99-1.38)	0.064	1.17 (0.90-1.53)	0.231					1.12 (0.96-1.31)	0.139	1.13 (0.82-1.57)	0.426
**Ever HIV tested**												
Yes	1.00	**0.032**	1.00	0.204					1.00	**0.040**	1.00	0.192
No	**0.60 (0.37-0.95)**		0.85 (0.59-1.22)						**0.65 (0.43-0.99)**		0.64 (0.33-1.27)	
**Pseudo R squares**												
Nagelkerke					0.08		0.07		0.12		0.12	

^§^Women with childbirth in the previous five years before and until 2007 were included in analysis.

Significant odds ratios and p values indicated in bold. Odds ratios calculated by multivariate logistic regression using complex samples. All confidence intervals (CI) adjusted for clustering effect using SPSS Windows Version 19 with the standard enumeration area (SEA) as clusters.

†The number in brackets indicates number of levels from lowest to highest for covariates that were entered in the model as continuous variables.

**Table 4:** **Bivariate and multivariate logistic regression of health facility childbirths adjusted by socio-economic, socio-demographic and proximate factors in Malindi district, Kenya in women aged 15–49 years (N = 1800)**^**§**^

	**Bivariates**				**Multivariate step 1**				**Multivariate step 2 (full model)**			
	**Rural**		**Urban**		**Rural**		**Urban**		**Rural**		**Urban**	
	**OR (95% CI)**	**p value**	**OR (95% CI)**	**p value**	**AOR (95% CI)**	**p value**	**AOR (95% CI)**	**p value**	**AOR (95% CI)**	**p value**	**AOR (95% CI)**	**p value**
**Underlying factors**												
**SEP (1–16)†**	1.09 (0.99-1.20)	0.071	**1.16 (1.09-1.23)**	**0.001**	1.09 (0.99-1.20)	0.075	**1.16 (1.09-1.23)**	**0.001**	1.06 (0.96-1.17)	0.228	**1.14 (1.09-1.16)**	**<0.001**
**Age (15–49)**	0.96 (0.93-1.00)	0.066	1.02 (0.92-1.09)	0.422	0.96 (0.92-1.00)	0.049	1.01 (0.96-1.07)	0.553	0.96 (0.92-1.01)	0.102	0.99 (0.94-1.05)	0.895
**Marital status**												
Married	1.00	0.486	1.00	0.386	1.00	0.530	1.00	0.314	1.00	0.395	1.00	0.166
Single/Divorced	0.77 (0.36-1.66)		1.32 (0.66-2.62)		0.79 (0.37-1.70)		1.45 (0.63-3.37)		0.70 (0.28-1.77)		2.03 (0.68-6.03)	
**Proximate factors**												
**Trust-Quality (1–4)**	1.02 (0.76-1.38)	0.885	1.03 (0.76-1.39)	0.834					1.04 (0.76-1.43)	0.804	1.00 (0.73-1.36)	0.962
**Perceived cost (1–5)**	0.73 (0.49-1.07)	0.097	**0.69 (0.48-0.98)**	**0.041**					0.97 (0.69-1.38)	0.942	0.71 (0.41-1.26)	0.191
**Perceived distance (1–5)**	**0.55 (0.41-0.73)**	**<0.001**	0.89 (0.55-1.44)	0.607					**0.58 (0.45-0.75)**	**<0.001**	1.09 (0.63-1.89)	0.686
**ANC visits (0–9)**	**1.32 (1.07-1.63)**	**0.013**	1.31 (0.95-1.81)	0.089					**1.29 (1.05-1.53)**	**0.018**	**1.35 (1.00-1.81)**	**0.050**
**Ever HIV tested**												
Yes	1.00	0.432	1.00	**0.011**					1.00	0.332	1.00	0.085
No	0.83 (0.51-1.35)		**0.58 (0.39-0.85)**						0.84 (0.57-1.24)		0.64 (0.37-1.12)	
**Pseudo R squares**												
Nagelkerke					0.04		0.15		0.18		0.22	

^§^Women with childbirth in the previous five years before and until 2007 were included in analysis.

Significant odds ratios and p values indicated in bold. Odds ratios calculated by multivariate logistic regression using complex samples. All confidence intervals (CI) adjusted for clustering effect using SPSS Windows Version 19 with the standard enumeration area (SEA) as clusters.

†The number in brackets indicates number of levels from lowest to highest for covariates that were entered in the model as continuous variables.

**Table 5:** **Bivariate and multivariate logistic regression of health facility childbirths adjusted by socio-economic, socio-demographic and proximate factors in Mbarali district, Tanzania in women aged 15–49 years (N = 1800)**^**§**^

	**Bivariates**				**Multivariate step 1**				**Multivariate step 2 (full model)**			
	**Rural**		**Urban**		**Rural**		**Urban**		**Rural**		**Urban**	
	**OR (95% CI)**	**p value**	**OR (95% CI)**	**p value**	**AOR (95% CI)**	**p value**	**AOR (95% CI)**	**p value**	**AOR (95% CI)**	**p value**	**AOR (95% CI)**	**p value**
**Underlying factors**												
**SEP (1–16)†**	**1.11 (1.01-1.22)**	**0.028**	**1.10 (1.00-1.21)**	**0.050**	**1.11 (1.01-1.22)**	**0.024**	**1.10 (1.01-1.20)**	**0.043**	**1.14 (1.03-1.26)**	**0.009**	1.09 (0.99-1.20)	0.110
**Age (15–49)**	1.00 (0.96-1.05)	0.898	**0.96 (0.93-0.99)**	**0.017**	1.00 (0.96-1.04)	0.855	**0.96 (0.94-0.99)**	**0.021**	1.00 (0.96-1.05)	0.915	0.98 (0.94-1.03)	0.365
**Marital status**												
Married	1.00	0.375	1.00	0.991	1.00	0.433	1.00	0.766			1.00	0.712
Single/Divorced	2.61 (0.29-23.27)		1.01 (0.20-5.00)		2.34 (0.26-20.75)		0.80 (0.17-3.80)				1.63 (0.11-24.97)	
**Proximate factors**												
**Trust-Quality (1–4)**	0.92 (0.67-1.26)	0.598	**1.58 (1.18-2.11)**	**0.004**					0.85 (0.57-1.28)	0.428	**1.63 (1.21-2.19)**	**0.003**
**Perceived cost (1–5)**	0.97 (0.77-1.21)	0.759	**1.58 (1.15-2.16)**	**0.007**					1.10 (0.83-1.47)	0.501	**1.65 (1.15-2.37)**	**0.009**
**Perceived distance (1–5)**	0.85 (0.69-1.04)	0.112	1.06 (0.76-1.47)	0.723					0.88 (0.70-1.12)	0.296	1.00 (0.67-1.50)	0.998
**ANC visits (0–9)**	1.08 (0.96-1.21)	0.191	1.13 (0.99-1.29)	0.066					1.08 (0.93-1.26)	0.282	1.11 (0.90-1.37)	0.327
**Ever HIV tested**												
Yes	1.00	0.133	1.00	**<0.001**					1.00	**0.028**	1.00	**<0.001**
No	0.65 (0.37-1.15)		**1.14 (0.42-3.08)**						**0.52 (0.29-0.92)**		**0.27 (0.17-0.43)**	
**Pseudo R squares**												
Nagelkerke					0.04		0.05		0.11		0.23	

^§^Women with childbirth in the previous five years before and until 2007 were included analysis.

Significant odds ratios and p values indicated in bold. Odds ratios calculated by multivariate logistic regression using complex samples. All confidence intervals (CI) adjusted for clustering effect using SPSS Windows Version 19 with the standard enumeration area (SEA) as clusters.

†The number in brackets indicates number of levels from lowest to highest for covariates that were entered in the model as continuous variables.

#### Facility childbirth and SEP

In the bivariate analysis SEP was positively associated with facility childbirth in all districts except from a borderline significance in rural Malindi (Tables 3, 4 and 5). The strength of the associations did not change substantially when adjusting for the other model variables, but the associations in rural Malindi and urban Mbarali appeared borderline significant. The strength of association might best be expressed by estimating the likelihood of facility childbirth amongst those with the lowest vs. highest value of SEP. This comparison of the extreme levels of SEP in rural areas showed the adjusted odds ratio (AOR), 2.35 in Malindi, 7.58 in Mbarali and 7.03 in Kapiri Mposhi. Similarly, the AOR was 8.67, 3.32 and 4.83 in urban areas of the three districts respectively.

### Facility childbirth with associated proximate factors

Perceived quality was not associated with health facility childbirth in Malindi and Kapiri Mposhi, whereas a positive association was observed in urban Mbarali, AOR 4.34 comparing extremes of perceived quality-trust. Local variation was also observed with association of perceived cost and facility childbirth. The negative association observed in urban Malindi was insignificant after adjusting for underlying and proximate factors. In contrast, a positive association was observed in urban Mbarali and Kapiri Mposhi (Tables 4, 5 and 3). Perceived distance tended to reduce the likelihood of facility childbirth in urban areas of Kapiri Mposhi, and in rural Malindi. ANC visits tended to be positively associated with facility childbirth in Malindi district, i.e. statistically significant or borderline significance. Women who reported ever being HIV tested were also more likely to have facility delivery compared to those never tested, and the association remained significant in both rural and urban Mbarali and rural Kapiri Mposhi in the final model (Tables 5 and 3).

The proportion of variance accounted for, an approximation using Nagelkerke R square, differed by district. In Malindi, the proximate factors contributed a greater proportion in the rural area, whereas in the urban area, SEP contributed to a greater extent than the proximate factors. In Mbarali, and particularly in the urban areas, the proximate factors had greatest contribution. Nagelkerke R square value was small regardless of residence and with equal contribution of SEP and proximate factors in Kapiri Mposhi.

## Discussion

There were substantial differences in the proportion of health facility childbirth among the districts and particularly when comparing rural areas. However, striking socio-economic inequities were revealed regardless of district in addition to geographical inequities in two districts. Furthermore, there were indications that repeated exposure to ANC services and to HIV related counselling and testing was positively associated with health facility deliveries. Perceived distance as a barrier to health facility childbirth was most consistent in rural areas, whereas association with perceived direct cost at the facility differed by place. Perceived quality and trust of local health services showed significance in urban Mbarali, whereas age of the mother did not seem to be associated with place of delivery.

Pregnant women in rural areas and in groups with low socio-economic position have been shown to be at increased risk of maternal mortality [47], thus presenting a special need for targeted health services during childbirth among these women who are less likely to utilize the health services. Women from higher SEP are more likely to make informed decisions as to place of delivery, demand better services, have financial accessibility, and more likely to live in urban areas where facilities are nearby [13,14,40,42,46,48]. Improved SEP of women is likely to reduce maternal and neonatal morbidity and mortality through changes in health seeking behaviour due to acquired knowledge through education and improved access to health services. This study showed both educational attainment and wealth index to be positively associated with giving birth at a health facility. Improving the educational attainment of women is an example of a structural intervention with long-term effects. In Sri Lanka, national policies promoting female education coupled with widespread provision of health services to the poor were seen as some of the key factors that influenced women to use healthcare services [49].

Wealth index based on household assets was used as the second indicator of SEP in this study. The use of household asset ownership in constructing wealth index has been shown to produce less measurement error compared to consumption expenditure or income in low income countries. Asset ownership uses simple questions and direct observation by the interviewer and has less recall and social desirability biases when obtaining information [50]. Household asset ownership also represents long-term SEP and unlikely to be changed by short-term economic shocks [50]. However, an obvious weakness of using household assets is the overriding role of access to electricity, and thus urban households are given substantially higher scores than those in rural settings due to the geographical difference in access to electricity. Our strategy to reduce this weakness was to stratify our analysis by residence.

Perceived cost was associated with increased likelihood of facility childbirth in urban areas of Mbarali and Kapiri Mposhi. In both these areas childbirth services were offered free of charge in public health facilities at the time of the study. It might be possible that women either by-passed fee-charging facilities or preferred them because they offered better quality care. In this study private health facilities were not utilized in Kapiri Mposhi whereas in urban Mbarali more women utilized mission or non-governmental health facilities. Here facility childbirths were observed to be positively associated with trust and perceived quality of care. This compares with a study in Tanzania that described the phenomenon of by-passing health facilities in preference for better quality of care [44]. These differences may indicate complexities in care seeking behaviour at childbirth when women consider cost as well as quality of care. User fees in public health institutions were abolished for pregnancy and childbirth in Zambia in 2006, and free maternal deliveries introduced in Kenya from 2007, whereas Tanzania has practiced free services from around 2000 [51]. Healthcare financing by government as a percentage of total health expenditure has been observed to differ across the countries, with Kenya, Zambia and Tanzania spending 43%, 59% and 66%, respectively in 2009 [52]. The share of healthcare financing by government has been shown to be associated with increased utilization of skilled attendance at birth [53]. This could be due to increased access to maternal health care services and less out-of-pocket expenses with government compared to private sector financing [53].

Despite free maternal health services, the walking distance, cost of and lack of transport, and poor road infrastructure especially in rural settings make it difficult for women to decide to use or reach a health facility during childbirth [41]. Perceived distance was associated with reduced facility childbirth in rural Malindi and has been observed in other studies [13,16-18]. In sub-Saharan Africa, majority of individuals live in rural areas which have long distances to health facilities and low coverage of skilled attendance [54,55]. Unfortunately, perceived distance is also a barrier in the urban poor. Our full model showed perceived distance to be a barrier in urban Kapiri Mposhi.

Assessing perceived quality of care by the community is important to determine users’ perspectives of functioning of the health system and improve responsiveness of care providers [56]. Quality assessed by availability of emergency obstetric care services, adequate staff with proper skills, provider attitudes to patients, and adequate drugs and supplies influenced use of health care services in Tanzania [46,57,58]. We found a positive association of perceived quality of care and facility childbirth in Mbarali but not in Malindi and Kapiri Mposhi. Previous study in Zambia also found no association of perceived quality of care and use of health professionals for childbirth [17]. A possible explanation is that measurement of perceived quality of health care in low income countries is subject to social desirability bias as respondents tend to give a more favourable response [59]. However, studies in Guinea and India showed high reliability for components of health personnel attitudes for the measure of quality of health care [60-62].

The number of ANC visits tended to be positively associated with giving birth at a health facility, although statistically significant only in Malindi. Other studies have shown similar relationship for ANC attendance [16]. Being tested for HIV was also positively associated with facility childbirth, with statistical significance observed in Mbarali and rural Kapiri Mposhi. Use of healthcare services such as ANC and HIV related counselling and testing could reflect the positive impact of counselling by health providers to influence change in health seeking behaviour. Interaction of women with health services over time may help women acquire knowledge about safe delivery and make them more comfortable with professional health care. The provider-initiated opt-out HIV testing model has been practiced in PMTCT programmes. A previous mixed method study from the same three districts found that counselling related to HIV testing was highly valued, but that the opt-out model had resulted in a drastic reduced focus on counselling as compared to the voluntary HIV counselling and testing model [30]. This raises concerns of how the consent process is taken care of and that many preventive opportunities are missed out, e.g. that HIV negative persons are not provided with HIV preventive counselling [30]. This is very unfortunate since PMTCT programmes have now almost universal coverage. Hence they are offering a great opportunity for more integrated models for prevention, e.g. by also including safe delivery counselling. Research is urgently needed to evaluate effects of such integrated models putting high counselling focus.

The AFR approach, in an effort to reduce gaps in achieving trust, quality and equity, is seen as feasible from other studies, although a number of constraints were also identified [63]. This approach could help reduce gaps in access to and the use of health services, although with possible challenges, by setting priorities that are fair, legitimate and accountable to needs of communities. Deliberate efforts to reduce inequities could be achieved by policies that target and involve disadvantaged groups in order to approach equal access to health care for equal need, and equal utilisation for equal need. Such measures could improve skilled birth attendance and pregnancy outcomes.

This study adds to the knowledge of existence of socio-economic inequities in rural and urban areas as major underlying factors in health facility utilisation at childbirth in these three sub-Saharan countries. It is not clear to what extent our findings can be generalised to other districts in the three countries. Initially the three districts were selected due to similarities in disease burden as well as district health system structure. However, they were found to differ substantially in the public-private composition and health care seeking in general. This reflects both health system and cultural differentials which are likely to shape skilled birth attendance as revealed in the substantial inter-district differences. Consideration of context is of critical importance in formulating interventions in order to provide accessible, equitable and quality services. Limitations of this study could include measurement bias regarding perceived quality of care, distance and cost. It is possible that some participants could over-rate the services because they fail to openly criticize the system [59,60]. However, perceptions give the user’s perspectives of health system functioning and provides opportunities for improvement of service provision [56]. Although these biases may have been present, they may not have been important factors explaining major observations.

## Conclusion

Strong socio-economic inequities in the likelihood of facility childbirths were revealed in all the districts. Geographic inequities were revealed in Malindi and Kapiri Mposhi. These findings suggest an urgent need to strengthen services targeting disadvantaged populations. In addition, potential positive effects can be achieved by strengthening integrated approaches in maternal health service delivery as suggested by a positive association between HIV counselling/testing and facility childbirths. Furthermore, setting specific factors of physical access and cost that are associated with facility childbirth need to be addressed.

## Competing interests

The authors declare that they have no competing interests.

## Authors’ contributions

SNP was involved in conceptualising, analysing the data, interpreting the findings, literature search and writing the manuscript. GK and TK were involved in conceptualising the objective of this paper, interpreting findings and revisions of the manuscript. JB was involved in conceptualising the REACT project, development of the standard operating procedures and revising the manuscript. CM was involved in questionnaire development, development of standard operating procedures and revisions of this manuscript. BEO was involved in development of modules related to maternal health and obstetric care used in data collection, and in revisions of manuscript. EE was involved in data collection, drafting and critically revising this manuscript. KF was involved in conceptualising the REACT project, development of the questionnaire and the standard operating procedures, and also in conceptualising the objective, analysing and interpreting the data, and revised the manuscript. All authors approved the final version.

## Pre-publication history

The pre-publication history for this paper can be accessed here:

http://www.biomedcentral.com/1471-2393/14/219/prepub
